# A home-based rehabilitation intervention for people living with HIV and disability in a resource-poor community, KwaZulu-Natal: study protocol for a randomised controlled trial

**DOI:** 10.1186/s13063-015-1025-2

**Published:** 2015-11-02

**Authors:** Saul Cobbing, Jill Hanass-Hancock, Hellen Myezwa

**Affiliations:** Department of Physiotherapy, University of KwaZulu-Natal, Private Bag X 54001, Durban, 4000 South Africa; Health Economics and HIV/AIDS Research Division (HEARD), University of KwaZulu-Natal, Durban, South Africa; Department of Physiotherapy, University of the Witwatersrand, Johannesburg, South Africa

**Keywords:** HIV, Home-based rehabilitation, ICF, Task shifting

## Abstract

**Background:**

In the era of highly active antiretroviral therapy HIV is now viewed as a chronic disease. Although people living with HIV are living longer lives, they are prone to a number of disabilities. Home-based rehabilitation has been shown to be an effective means of improving quality of life and function for people with a wide range of chronic diseases. There is a dearth of evidence, however, related to home-based rehabilitation interventions for people living with HIV, particularly in sub-Saharan Africa — the region with the highest global prevalence of HIV.

**Methods:**

A randomised controlled trial design will be employed. Adults living with HIV who have been on antiretroviral therapy for at least six months and with defined limited mobility will be randomly allocated to either an intervention group or the control group. Pre and post-intervention testing will be conducted at a public hospital in KwaZulu-Natal, South Africa in order to assess the participants’ quality of life, perceived level of disability, functional ability and endurance. Individuals randomly allocated to the intervention group will participate in a four-month home-based rehabilitation programme, conducted once a week in their homes. This programme will be implemented by community workers who will be trained and supervised by a qualified physiotherapist. The participants in the control group will continue with the standard clinic management offered to them. On completion of the intervention, all participants will be re-assessed using the same outcome measures. Analysis of results will be carried out on intention-to-treat basis in order to identify any changes between intervention and control groups.

**Discussion:**

The researchers aim to employ a novel task shifting approach to implement a needs-based home-based rehabilitation programme for people living with HIV in order to improve their quality of life and functional ability. It is hoped that this study will provide rehabilitation professionals and researchers with evidence that can be utilised to improve existing rehabilitation interventions for people living with HIV.

**Trial registration:**

South African National Clinical Trials Register: NHREC#4094 (Date of registration: 21 July 2015).

## Background

Globally, it is estimated that there are 35.3 million people living with HIV (PLHIV) with 25 million PLHIV in sub-Saharan Africa. An estimated 6.1 million people are currently living with HIV in South Africa [1], more than in any other single country in the world. Within South Africa itself, KwaZulu-Natal (KZN) has the highest HIV prevalence of all the country’s nine provinces, with 14.8 % of its inhabitants living with HIV. With improved treatment options for PLHIV, specifically the advent of highly active antiretroviral treatment (HAART), HIV is now viewed as a chronic disease rather than a terminal illness [2]. As an illustration of this, life expectancy in 2011 in KZN province was 11.3 years higher than in 2003, when the scale-up of HAART for PLHIV was in its infancy [1].

Although they are now living longer due to HAART, PLHIV are also more prone to a number of impairments and disabilities that may require rehabilitation. These disabilities may be permanent (such as blindness) or episodic in nature, with the extent of an individual’s impairments (and their experience of these impairments) fluctuating over time, depending on a number of inter-related factors [3]. A recent qualitative paper on the experiences of disability in PLHIV concludes that future HIV research needs to consider the new health care needs of PLHIV beyond a focus on clinical conditions and address the activity and participation restrictions that come with a life on HAART [4]. Rehabilitation services can help PLHIV address the life-related consequences of their medical condition [2] and can enhance their daily functioning via a number of strategies, including exercise [5].

Exercise and rehabilitation have been shown to be beneficial for PLHIV in a number of different settings [6, 7]. However, there is a great disparity in access to rehabilitation for PLHIV between developed and developing countries [8]. The current model prevalent in the South African public health system favours rehabilitation of patients in hospitals or clinics, despite evidence of the urgency to raise the profile of community-based rehabilitation (CBR) in South Africa at national, provincial and district levels [9]. This shortfall of widespread CBR provision is contrary to the South African Department of Health’s [10] vision of public health care delivery in South Africa moving towards a model that provides rehabilitation services in or near to patients’ homes. There is extensive evidence supporting the effectiveness of home-based rehabilitation (HBR) interventions for other chronic disease populations, including stroke [11–13] and coronary artery disease [14, 15]. However, there is a dearth of literature describing sub-Saharan African HBR interventions in general and no studies that have investigated HBR programmes designed specifically for PLHIV and disability in South Africa.

In the resource-poor community where this research is being conducted, PLHIV have identified a number of barriers to accessing the traditional institution-based physiotherapy rehabilitation available to them. These barriers include cost of rehabilitation, transport and accessibility issues [16]. This preliminary research has demonstrated the need for alternative evidence-based rehabilitation interventions for PLHIV in South Africa, particularly those living in resource-poor environments. The authors hypothesise that this HBR programme designed specifically for PLHIV and disability will have a positive effect on participants’ perceived disability, quality of life, functional mobility and functional capacity and will thus offer an effective alternative to institution-based rehabilitation for this patient population.

### Research question

What is the effect of a needs-based HBR intervention on PLHIVs’ perceived disability, quality of life, functional mobility and functional capacity?

### Aim

To determine the effect of a needs-based HBR intervention on PLHIVs’ perceived disability, quality of life, functional mobility and functional capacity.

### Objectives

1. To design a needs-based HBR intervention for adult PLHIV in a resource-poor setting using a task shifting approach.
2. To determine the effects of the HBR intervention on participants’ perceived disability.
3. To determine the effects of the HBR intervention on participants’ quality of life.
4. To determine the effects of the HBR intervention on participants’ functional mobility.
5. To determine the effects of the HBR intervention on participants’ functional capacity.

### Conceptual framework

**Fig. 1:**
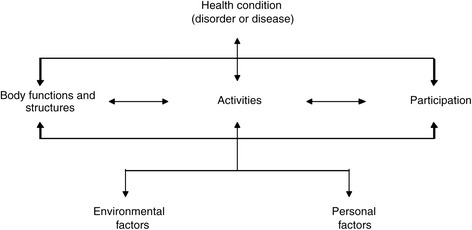
Graphic representation of the ICF framework

This study uses the International Classification of Functioning, Disability and Health (ICF) as a guiding framework. The ICF [17] offers a useful framework for studying disability and health-related consequences of disease such as HIV [18]. This framework is based on three concepts: body function and structure, activity levels and participation. Changes in body function or structure (also named impairments) are understood to be problems with physiological functioning or anatomical (for example organs, limbs) structures of the body. Similarly, activity limitations are defined as difficulties in executing a task or action, such as walking or dressing. Finally, participation restrictions are problems relating to involvement in life situations [17]. Disability can be experienced at any of these levels and further influenced by inter-related environmental and personal factors (see Fig. 1). Thus, two individuals with the same impairment (for example, pain secondary to an HIV-related opportunistic infection) may experience markedly different activity limitations and participation restrictions, due to their respective socio-economic environments and specific personal characteristics. The authors of this study have utilised this framework in the design of this study in order to better reflect the overall lived experience of the participants who are exposed to the rehabilitation intervention.

## Methods

### Study design

A randomised controlled trial (RCT) design will be used in this study in order to assess the effectiveness of a 16-week HBR intervention designed specifically for PLHIV and disability. The RCT is considered the highest level of evidence when attempting to establish the effectiveness of a chosen intervention [19].

### Study area

The study is located at a public hospital in the eThekwini district of the province of KwaZulu-Natal, South Africa. This hospital serves a population of 750,000 people in Mariannhill, which is an impoverished rural and peri-urban area on the outskirts of Durban. The hospital and its outlying clinics provide a service for more than 4,500 PLHIV [20]. The eThekwini district is one of the ten municipal districts in South Africa with the highest HIV prevalence, with an HIV prevalence of 38 % amongst expectant mothers [21]. While the assessment of patients pre- and post-intervention will be conducted on the hospital premises, the intervention itself will be conducted in, or near to, participants’ homes. These communities fall within the hospital catchment area, which is defined as including individuals living within the Inner and Outer West Operational Entities of the Durban Metropolitan Area, a large geographical area spanning approximately 40 kilometres [20].

### Study population

Participants for the proposed study will be recruited from the HIV-LIVE study, a large cohort study that included 1,043 PLHIV who receive HAART from clinics that fall under the study hospital described above. The HIV-LIVE study is an ongoing collaboration between the Health Economics in HIV/AIDS Research Division (HEARD) and the University of the Witwatersrand’s Department of Physiotherapy, which aims to determine the extent and impact of HIV-related disability on PLHIV in South Africa. It is a cohort study that employed a cross-sectional design and includes, as one of many assessment measures, the short form of the World Health Organisation Disability Assessment Schedule (WHODAS 2.0) assessment tool, a self-reported measure of disability [22].

### Study sample and recruitment

Individuals from the study population who meet the inclusion and exclusion criteria (see Table 1) will be contacted by telephone and invited to the study hospital. On their arrival, the study will be described in detail to each individual (in their home language—most of the study population speak isiZulu), each prospective participant will be given a study information sheet and they will be asked to sign an informed consent form, should they agree to participate in the study. Baseline blood pressure and heart rate measures will also be taken to ensure that each individual is fit to complete the pre-intervention testing. General questions about their current health will also be asked to ensure that individuals with acute illnesses are not exposed to further risk of testing. On agreeing to participate, all participants will continue to complete the four study outcome measures. Ethical approval for both the main HIV-LIVE study and this RCT has been given by the South African Department of Health, University of KwaZulu-Natal (UKZN) and the University of Witwatersrand.

**Table 1:** Study inclusion and exclusion criteria

*Inclusion criteria*
Males and females living with HIV attending an outpatient HIV clinic of the study hospital
Individuals who have received HAART for six months or longer, specifically one of the national HAART regimens for adults, as outlined by the South African Department of Health.
Individuals over the age of 18
Individuals who scored for limitation in the mobility domain according to the WHODAS 2.0 (12 item version) assessment tool.
*Exclusion criteria*
Pregnant or breast-feeding women.
Any individual with an acute AIDS-defining opportunistic infection.
Any individual with a complete spinal cord injury with no possibility of being able to walk.
Unstable angina, recent myocardial infarction and elevated blood pressure (as per contraindications for the Six Minute Walk Test)

### Sample size

The sample size for this study has been calculated based on the group of participants from the HIV-LIVE cohort study who scored for limitation in the mobility-related questions (S1 and S7) of the WHODAS 2.0 (12 item version) tool. The effect size was chosen for these parameters. This was set at an alpha level 0.05 and allowed for a 40 % drop-out rate as indicated in previous studies (power of 80 %). A sample size of 80 participants is required, based on these parameters.

### Testing procedure

Five lay health workers, currently working in the study area for a non-governmental organisation, will conduct the pre-intervention testing, over a period of one week, at the study hospital. They will explain the nature and requirements of the study and provide each prospective participant with an additional study information sheet. Each individual will then be invited to participate in the study and, if they agree, asked to sign the consent form, in the full understanding that they be permitted to withdraw from the study at any point. Thereafter participants will undergo the pre-intervention testing. The four baseline outcome measures will each be conducted by a separate individual. The participants will be interviewed in their first language using a questionnaire including the short version of the World Health Organisation Quality of Life assessment tool (WHOQOL-HIV BREF) [23] and the short form of the World Health Organisation Disability Assessment Schedule (WHODAS 2.0). All interviews will be conducted in a private location, ensuring confidentiality and comfort for the participants. Following the interviews, the individual participant’s functional ability will be assessed using the Rivermead Mobility Index (RMI). After a five minute rest, the participant will then be invited to complete the Six Minute Walk Test (6MWT). Participants’ heart rate and blood pressure will be assessed before and after the test using an ambulatory wrist sphygmomanometer. Following the test, participants will be instructed to sit and rest for five minutes and offered water, after which time they will return home.

### Outcome measures

The WHOQOL-HIV BREF contains brief demographic questions, followed by 31 questions related to the individual’s perceived quality of life. The short form of the WHODAS 2.0 tool consists of 12 core questions assessing an individual’s perceived level of difficulty in carrying out certain day-to-day activities and participating in social, occupational and educational environments. The RMI is a 15-item assessment tool involving a combination of self-reported and directly observed measures of function, such as transferring from a bed to a chair and walking up and down four steps. Although initially designed to assess the functional ability of stroke patients, it is appropriate for populations with a range of physical disabilities [24]. The 6MWT is a test designed to assess distance walked over six minutes as a submaximal test of aerobic capacity/endurance [25]. The 6MWT will be performed, according to the test standards, indoors along a flat, hard surface in a hall on the study hospital’s premises. The walking course will be 20 metres in length, marked at every one metre. The turnaround points will be marked with cones. Absolute contraindications for the 6MWT include the following: unstable angina during the previous month and myocardial infarction during the previous month. Relative contraindications include a resting heart rate of more than 120, a systolic blood pressure of more than 180 mm Hg, and a diastolic blood pressure of more than 100 mm Hg [26]. Individuals who report absolute contraindications for the trial will not be considered for the trial. Individuals who present with any of the relative contraindications will have these measures taken twice more at 15-minute intervals, and if all of these readings are below the accepted limits, they will be able to continue with the trial. The four outcome measures employed in this study are described in more detail in Table 2.

**Table 2:** Study outcome measures

Measure	Description
RMI	Assesses functional mobility in gait, balance and transfers. Consists of 14 self-reported items and one direct observation item, with items progressing in difficulty.
6MWT	This test measures the distance that a patient can quickly walk on a flat, hard surface in a period of 6 minutes. It evaluates the global and integrated responses of all the systems involved during exercise.
WHODAS 2.0 (12 item)	This is a self-reported assessment instrument for health and disability, directly linked to the ICF, used across all diseases. This 12-item version focuses primarily on activities of daily living and participation.
WHOQOL-HIV-BREF	This self-reported instrument, adapted for PLHIV, examines an individual’s perceptions of his/her quality of life across six domains: physical, psychological, independence, social, environment and spirituality.

### Randomisation and follow-up of participants

Following the pre-intervention assessments, participants will be randomly assigned to either the intervention group or a control group (standard care). Computerised randomisation and stratification (according to gender) will be employed in order to ensure homogeneity between intervention and control groups. This will be achieved using the randomise list [RAND()] function in the Microsoft Excel (2013) software package [27]. This will also satisfy allocation concealment, thus ensuring that the investigators will be unaware of to which group each participant will be allocated [28]. In the week immediately following the 16-week HBR intervention, participants from both intervention and control groups will return to the study hospital. The same four outcome measures will be assessed at this follow-up visit in order to ascertain the change from the pre-intervention measures.

### HBR intervention

The lead author (SC), a qualified physiotherapist, has conducted the training of four lay health workers who were selected from a pool of people who currently live in the area and volunteer as home-based carers for a non-governmental organisation in the study community. The training was conducted over a one-month period and was designed to equip these four individuals with the theoretical and practical skills to conduct the exercise and rehabilitation intervention at the homes of the participants randomised to the study intervention group. The HBR intervention will begin two weeks after the pre-intervention assessments to allow for the randomisation of participants and communication with each participant in the intervention group in order to set up a time for the first visit. The four health workers will work in pairs, with each pair being responsible for the rehabilitation of half of the participants in the intervention group. The justification for working in pairs is to ensure that participants will continue to be seen throughout the 16-week intervention, even if one of the health workers should be unavailable. Furthermore, it is often necessary for two individuals to assist a participant, particularly with mobility exercises such as learning to walk with crutches.

On the first home visit, SC will assess each participant and teach them strength and stretching exercises suitable for each individual. Upper limb and lower limb strength exercises will primarily be performed using MSD-Band [29] resistive bands (lightweight latex bands of varying resistance), which will be provided for each participant. Stretching exercises for the upper and lower limbs, trunk and neck will also be taught. Functional exercises, such as sitting to standing and bridging, will also be demonstrated where appropriate. All participants will also be encouraged, if able, to carry out a progressive walking programme over the 16-week period. Following this first visit, SC will fulfil a supervisory role, advising and helping the lay health workers if they have particular concerns or difficulty with any specific participant.

Following the first assessment week, when participants will begin their individualised HBR programmes, each participant will be seen in their own home once a week by their designated pair of health workers. Each appointment will be made by mobile phone call, and participants will be seen on a day and at a time convenient to them. While the majority of the participants in the intervention group are unemployed, home visits will also be scheduled for Saturdays to accommodate those participants who do work full-time. The stretching exercises will be performed at each visit, while the volume and resistance of the strengthening exercises will be progressively increased, as will the distance walked at each visit. Where possible, walking will be performed on the streets surrounding the participants’ homes, with each participant being accompanied by at least one health worker in order to ensure safety. Walking aids in the form of walking frames, elbow crutches and walking sticks will be provided to those participants who require them, following thorough assessment and instruction in their use by SC. Each participant will also be instructed on the specific home exercises they should do for the days between each weekly visit.

At each weekly visit, the health workers will complete a comprehensive entry into each participant’s individual diary, including information on the rehabilitation completed on that visit. The exercises done over the previous week, any equipment issued (such as resistive bands or walking aids) and any illness or problems that the participant may have had in the previous week will also be recorded. Should a participant not have been available to be seen at home on any given week, the reason for this will be noted in the diary and the above information will be elicited via telephone conversation and recorded in the diary. At the halfway mark of the intervention (after 8 weeks) the participants will be requested to select a date and time for their post-intervention assessment at the study hospital. They will be reminded of this follow-up appointment in the last (16th) week of the HBR intervention.

### Control group

The participants randomised to the control group will receive the standard of care treatment offered by the specific clinic where they receive their HAART medication. They will be given a two-page information sheet on healthy living for PLHIV and encouraged to follow this advice. In the event that they are referred for rehabilitation by their clinic nurse or doctor, this will be conducted by a qualified physiotherapist at the study hospital’s physiotherapy department. Participants in the control group will receive a monthly phone call from one of the four lay health workers involved in the HBR intervention. These phone calls will serve to determine their health status and whether their contact/address details have changed. The phone call in the last month will also be utilised to set up a date and time for their post-intervention assessment at the study hospital. On completion of this study, should the HBR intervention be found to be effective, participants in the control group will be offered the opportunity to receive this intervention, at no cost to themselves.

### Blinding

The four lay health workers who will be involved in the HBR intervention are different individuals than the five lay health workers who are employed to conduct the pre- and post-intervention assessment. These latter five individuals will be blinded (at the post-intervention assessment) to the group allocation of participants in order to limit bias. Due to the nature of the intervention, double blinding was impossible, as participants will know whether they are receiving the intervention or are in the control group.

### Ethical considerations

1. Permission to conduct the study was obtained from the chief executive officer of the study hospital.
2. Ethical approval from the University of Kwazulu-Natal’s Biomedical Research Ethics Committee (BFC052/15) was received on 11 September 2014.
3. This study is registered with the South African National Clinical Trial Register (SANCTR); registration number: NHREC# 4094 as well as the South African Department of Health (DOH number: DOH-27-0715-5094).
4. Participants will be given an information sheet outlining the nature and requirements of the study and their completely voluntary participation, with the right to withdraw at any point during the study. Following this, they will be required to sign a consent form to participate in the study.
5. During all assessments requiring verbal discussion, participants will be interviewed in private locations at the study hospital in order to maintain privacy and confidentiality.
6. A physician at the study hospital must be informed of when the Six Minute Walk Test assessments will be performed and immediately called should participants suffer any adverse response during or after the walk.
7. Any participants who require any medical assistance directly related to the rehabilitation intervention will be immediately referred to the study hospital for assessment and treatment.
8. Participants in the control group will be offered the HBR intervention, should it prove to be beneficial.
9. While no inducements will be offered to participants, their transport costs to and from the study hospital will be covered. A small allowance for food will also be provided to participants for each of their two visits to the study hospital.
10. Participants will be assigned codes in order to maintain their anonymity. No information regarding their involvement in this study will be divulged at any point.
11. All information obtained from participants will be kept under lock and key and electronically on a password protected computer at the UKZN department of physiotherapy for 5 years, after which time all study data will be destroyed or deleted.

### Data analysis

Statistical analysis of collected data will be performed with the assistance of a qualified statistician, employed by the UKZN School of Health Sciences. This statistical analysis will be performed on an intention-to-treat basis. This form of analysis requires participants to be included even if they did not fully adhere to the protocol. The rationale for this approach is that, in this study, it is important to estimate the effects of allocating an intervention in practice, not only the effects in the subgroup of the participants who adhere to it. Descriptive statistics, such as mean and mode, will be used to evaluate baseline demographic data. Analysis of changes between the intervention and control groups will be compared using analysis of variance (ANOVA) and/or analysis of covariance (ANCOVA), determined by the consulting statistician. Level of significance will be set at *P* ≤ 0.05 with confidence intervals set at 95 %. The Statistical Package for the Social Sciences (SPSS) version 22 will be employed to record and analyse the study data.

## Discussion

HIV is now viewed globally as a chronic disease rather than a terminal illness [30]. However, it was important that the lay health workers involved in the testing and implementation of this HBR intervention were cognisant of the specific challenges facing both PLHIV and those working with PLHIV. This is particularly important when considering the ethical issues around implementing an intervention designed for PLHIV. Despite the improvements in HIV treatment and care, HIV has always presented specific ethical challenges to scientists, patients and caregivers [31]. Using ethnographic material collected from five HIV clinics in South Africa, Uganda and Thailand, Heimar (2013) distinguishes between what is understood as official ethics (including the universal principles of autonomy, beneficence, non-maleficence and justice) and “ethics on the ground” [31]. This study has ensured that official ethical principles were followed (see the above 11 ethical considerations). However, the question remains: If participants have been fully informed and subsequently sign a consent form, have we done enough to meet our ethical obligations? We would argue that we need to do more and this is why, in addition to the considerations outlined above, the lay health workers underwent thorough ethical training before the pre-assessments and HBR intervention began. Research conducted across five South African samples from township, urban and clinic settings showed that stigmatisation of PLHIV in South Africa is widespread. For example, between 33 % and 56 % of respondents believed that PLHIV should expect some restrictions on their freedom [32]. In extreme cases, people in South Africa who have revealed their HIV status have been assaulted and killed [33]. It would be naïve, therefore, to assume that PLHIV in this study’s community will necessarily be treated the same as people who suffer from other chronic diseases, such as diabetes or asthma. In this respect, as part of their training, the lay health workers involved in this study have been requested to inform other community members, if asked, that the trial is investigating the benefits of home-based exercise for people living in the community. At no point will participants’ HIV status be divulged to anybody outside of the study. Furthermore, should any participant feel that the attendance of the lead author or the lay health workers at their homes may cause them any harm or distress, every effort will be made to continue to see them in an alternate location where they feel more at ease. In addition to this, we will strongly support the right of any participant to withdraw from the study at any time and for any reason.

Despite the ethical challenges of this particular study, it is important that a study of this nature is conducted in this community, an area with such a high prevalence of PLHIV. This HBR intervention is novel for a number of reasons. While two systematic reviews have shown that both resistance and aerobic exercise are safe and beneficial for PLHIV, most of the articles included in these reviews investigated exercise conducted at health institutions or public exercise facilities [6, 7]. A thorough review of the literature by the authors identified only six peer-reviewed publications that have investigated the effectiveness of home-based exercise programmes for PLHIV [34–39]. Of these studies, two employed case study designs [36, 38] and two included a combination of home and institution-based exercise [37, 38]. Furthermore, only three of these studies [37–39] were conducted in sub-Saharan Africa and none in KZN, the province with the highest HIV prevalence in South Africa, the country with the highest HIV prevalence in the world [1]. Of the three sub-Saharan African-based studies, only one [39] exclusively employed a home-based protocol, and this study specifically investigated the effects of a walking programme on PLHIV, without the inclusion of broader rehabilitation elements. Thus this study is, to the authors’ knowledge, the first RCT conducted in sub-Saharan Africa that is exclusively home-based in nature and designed to provide needs-based rehabilitation for PLHIV and disability.

The distinction between exercise and rehabilitation is an important one. The majority of published articles on RCT exercise interventions for PLHIV involve a set programme of strength (for example, dumbbell curls) and/or resistance exercises (for example, treadmill walking) over a set period of time that is strictly implemented regardless of the differing needs of each individual participant [6, 7]. Rehabilitation, although generally including exercise components, involves more than just exercise. Worthington et al. [30] define rehabilitation as a dynamic process involving all prevention and treatment activities or services that address physical impairments, activity limitations and participation restrictions for an individual. This implies that rehabilitation must be adapted to the specific needs of the individual and should further be refined and adjusted as the period of rehabilitation continues. This study again demonstrates its novelty by investigating a needs-based HBR intervention, where the activities and exercises performed by each participant will be suited to their individual needs and progressed at a pace specific to their ability and adaptation to the demands of the programme. Each participant’s rehabilitation programme will be based on the study pre-assessment period, which focuses on functional measures, the presentation of the participant at the first home visit, as well as ongoing assessment of the participant’s progress. While this type of intervention may be more difficult to describe and replicate, it will more closely mirror the interventions performed by therapists working in the field of rehabilitation of PLHIV. It is hoped that this will ensure that the results of this study are more applicable and translatable to therapists working with PLHIV in community settings, particularly in sub-Saharan Africa.

Another novel aspect of this study is the fact that it employs a task shifting approach in the design and application of this HBR intervention for PLHIV. Despite the high prevalence of HIV and disability in South Africa, there are relatively few trained physiotherapists working in this country (per capita population), compared to countries in the developed world. Recent WHO statistics [8] reveal that there are more than 20 physiotherapists per 10,000 population in Finland, as compared to less than two physiotherapists per 10,000 population in South Africa. With regard to another rehabilitation profession, occupational therapy, Denmark has approximately 11 occupational therapists per 10,000 population, compared to less than one occupational therapist in South Africa, per 10,000 population. This relative paucity of rehabilitation professionals requires alternative approaches, particularly in a country with such a high prevalence of PLHIV. One such alternative is to employ a task shifting approach, which can be defined as a “process whereby specific tasks are moved, where appropriate, to health workers with shorter training and fewer qualifications” [40], for example (as in this case), from physiotherapists to lay community care workers. This has been shown to be an effective strategy for addressing shortages of human healthcare resources in a number of areas of HIV care and treatment, including HAART management by nurses rather than doctors [41] and the provision of basic clinical tasks by lay health care workers [42]. A study conducted by one of the authors of this paper (in the same community) showed the potential for appropriately trained lay HIV counselors to carry out group-based counseling interventions for PLHIV and depression [43]. The success of this study has now been integrated in a current larger RCT trial in South Africa [44]. Additional opportunities related to task shifting for PLHIV include potential cost advantages, enhancing the role of the community and improving the efficiency of existing health systems [45]. For these reasons, and despite the ethical challenges inherent in this type of research, it is anticipated that this study will add new data and understanding to the field of rehabilitation for PLHIV and disability. It is hoped that this information will be of interest to a wide range of rehabilitation professionals and researchers, particularly those working in resource-poor communities, and that it will contribute to the design and implementation of improved rehabilitation options for this vulnerable population.

## Trial status

This trial is currently ongoing. Ethical approval from the UKZN Biomedical Research Ethics Committee, the South African National Clinical Trial Register (registration number: NHREC# 4094) as well as the South African Department of Health (DOH number: DOH-27-0715-5094) has been obtained. Recruitment of potential participants from the study population has begun.

## Abbreviations

6MWT: Six Minute Walk Test
AIDS: acquired immune deficiency syndrome
CBR: community-based rehabilitation
HAART: highly active antiretroviral treatment
HBR: home-based rehabilitation
HIV: human immunodeficiency virus
ICF: International Classification of Functioning, Disability and Health
KZN: KwaZulu-Natal
PLHIV: people living with HIV
RCT: randomised controlled trial
RMI: Rivermead Mobility Index
UKZN: University of KwaZulu-Natal
WHODAS 2.0: World Health Organisation Disability Assessment Schedule (version 2.0)
WHOQOL-HIV BREF: World Health Organisation Quality of Life (HIV) tool
